# Determination of the absolute bioavailability of oral imatinib using a stable isotopically labeled intravenous imatinib-d8 microdose

**DOI:** 10.1007/s00228-020-02888-y

**Published:** 2020-05-19

**Authors:** Jeroen Roosendaal, Stefanie L. Groenland, Hilde Rosing, Luc Lucas, Nikkie Venekamp, Bastiaan Nuijen, Alwin D. R. Huitema, Jos H. Beijnen, Neeltje Steeghs

**Affiliations:** 1 Department of Pharmacy & Pharmacology, Netherlands Cancer Institute – Antoni van Leeuwenhoek, Plesmanlaan 121, 1066 CX Amsterdam, The Netherlands; 2 Department of Medical Oncology and Clinical Pharmacology, The Netherlands Cancer Institute – Antoni van Leeuwenhoek, Amsterdam, The Netherlands; 3 Department of Clinical Pharmacy, University Medical Center, Utrecht University, Utrecht, The Netherlands; 4 Division of Pharmacoepidemiology and Clinical Pharmacology, Faculty of Science, Utrecht Institute for Pharmaceutical Sciences, Utrecht University, Utrecht, The Netherlands

**Keywords:** Imatinib, Microdosing, Absolute bioavailability, Stable isotope labeled, Steady state

## Abstract

**Purpose:**

The aim of this study was to ascertain whether the absolute bioavailability of oral imatinib (Glivec®) during steady state plasma pharmacokinetics in cancer patients could be determined through a concomitant intravenous administration of a single 100 μg microdose of deuterium labeled imatinib (imatinib-d8). Secondly, the usefulness of liquid chromatography–tandem mass spectrometry (LC-MS/MS) was investigated for simultaneous analysis of orally and intravenously administered imatinib.

**Methods:**

Included patients were on a stable daily dose of 400 mg oral imatinib prior to study participation. On day 1, patients received a 100 μg intravenous imatinib-d8 microdose 2.5 h after intake of the oral dose. Plasma samples were collected for 48 h. Imatinib and imatinib-d8 concentrations were simultaneously quantified using a validated LC-MS/MS assay. The absolute bioavailability was calculated by comparing the dose-normalized exposure with unlabeled and stable isotopically labeled imatinib in plasma.

**Results:**

A total of six patients were enrolled. All patients had a history of gastrointestinal stromal tumors (GIST). The median absolute bioavailability of oral imatinib at steady state was 76% (range 44–106%). Imatinib and imatinib-d8 plasma concentrations were quantified in all collected plasma samples, with no samples below the limit of quantification for imatinib-d8.

**Conclusion:**

The absolute bioavailability of imatinib was successfully estimated at steady state plasma pharmacokinetics using the stable isotopically labeled microdose trial design. This study exhibits the use of a stable isotopically labeled intravenous microdose to determine the absolute bioavailability of an oral anticancer agent in patients with LC-MS/MS as the analytical tool.

**Electronic supplementary material:**

The online version of this article (10.1007/s00228-020-02888-y) contains supplementary material, which is available to authorized users.

## Introduction

The last decade has shown an increasing number of anticancer drugs that are administered orally. [1–3] This so-called “intravenous to oral switch” in oncology has resulted in an increased attention on the investigation of the absolute bioavailability during clinical drug development. Determining the absolute oral bioavailability of a new drug candidate facilitates the identification of potential developmental challenges such as absorption and first pass metabolism during the clinical development of a drug. Hence, the assessment of the absolute bioavailability is also crucial for the development of optimized oral formulations. As a result, data on the absolute bioavailability of novel oral drugs is now increasingly requested by the FDA and EMA [4, 5].

The conventional way to assess the absolute oral bioavailability is by using a two-period crossover study design, where an intravenous dose and an oral dose are administered to a study subject with a washout period in between. The absolute bioavailability is then calculated by dividing the plasma exposure after oral administration by the plasma exposure after intravenous administration. A limitation of this design is that for drugs that are poorly soluble in aqueous media, it might be impossible to develop an intravenous formulation at therapeutic strength. In addition, it assumes linear pharmacokinetics and constant clearance between the oral and intravenous dose event, which might not always be the case for drugs demonstrating a high intra-patient variability. This may result in a systemic error in the determination of the absolute bioavailability. [6]

A study design of co-administering an intravenous isotopically labeled microdose (≤ 100 μg, less than 1/100th of the therapeutic dose) with a therapeutic oral dose provides a solution to these problems. Because only a small amount of drug needs to be dissolved in an intravenous formulation, drug solubility issues can be circumvented. In addition, according to the current regulatory guidelines, clinical intravenous microdose studies could be carried out without additional toxicity investigations, saving costs, and time associated with intravenous drug development. [7] Furthermore, because the intravenous microdose is administered during the same dose event as the oral therapeutic dose, the study duration is shortened and intra-occasion variability is not an issue, resulting in a more accurate determination of the absolute bioavailability and increased patients convenience.

Absolute bioavailability microdose trials can be performed using either radiolabeled or stable isotopically labeled drug processed into an intravenous formulation. In recent years, accelerator mass spectrometry (AMS) to measure a radiolabeled microdose has been utilized to support several clinical absolute bioavailability studies. [8] A drawback of AMS is that sample analysis is labor and time intensive, expensive, and that AMS is only available in a limited number of places dedicated to biomedical research worldwide. [9] An alternative analytical approach for conducting microdose studies is using liquid chromatography coupled to tandem mass spectrometry (LC-MS/MS) to quantitate both the intravenous and the oral drug. Because both labeled and unlabeled drug can be measured simultaneously with LC-MS/MS, it is an elegant and cost-effective alternative to AMS. [9, 10]

For the group of tyrosine kinase inhibitors, an important class of novel oral anticancer agents, it has been demonstrated that for many drugs registered in the past years, the absolute bioavailability has not been assessed at the time of drug licensing. [3] One reason for this might be that poor drug solubility hampers the development of an aqueous intravenous dose at therapeutic strength, making it almost impossible to use the conventional crossover trial design. In this trial, we used imatinib, a tyrosine kinase inhibitor used for the treatment of chronic myeloid leukemia (CML) and gastrointestinal stromal tumors (GIST), to demonstrate the potential of using a stable isotopically labeled 100 μg microdose in combination with LC-MS/MS to assess the absolute bioavailability.

The objective of this study was to ascertain whether the absolute bioavailability of oral imatinib (Glivec®) during steady state plasma pharmacokinetics in cancer patients could be determined through a concomitant intravenous administration of a single 100 μg microdose of deuterium labeled imatinib (imatinib-d8). Secondly, the usefulness of liquid chromatography–tandem mass spectrometry (LC-MS/MS) is investigated for simultaneous analysis of orally and intravenously administered imatinib.

## Materials and methods

### Study design and sample collection

**Fig. 1:**
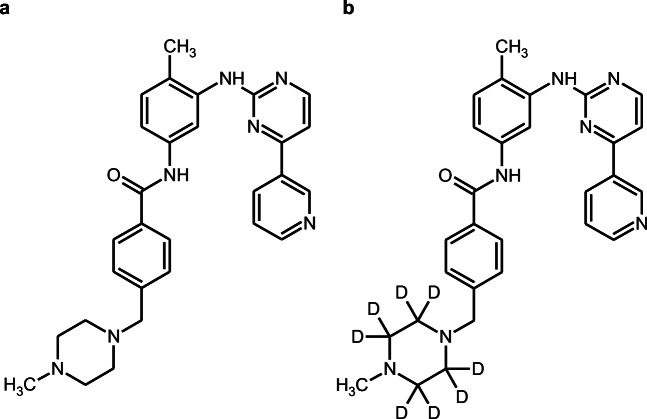
Molecular structures. **a** Imatinib. **b** Imatinib-d8

**Fig. 2:**
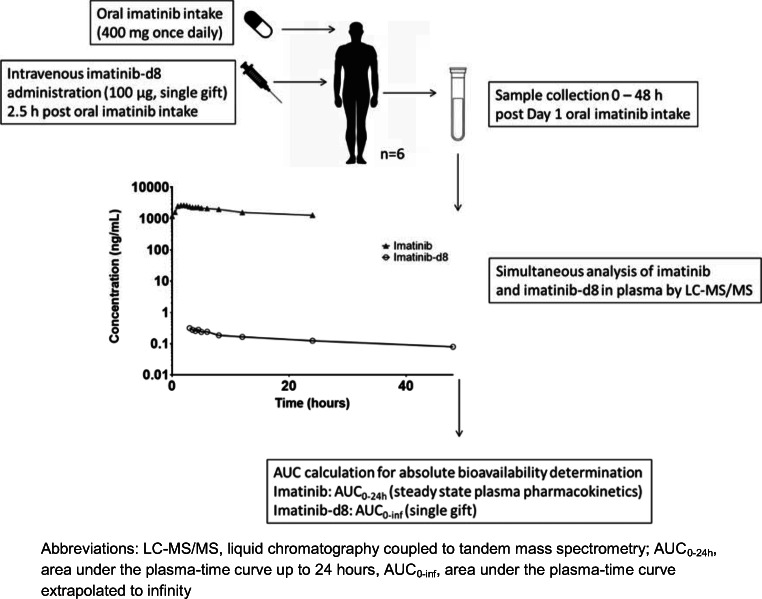
Schematic overview of the imatinib absolute bioavailability microdose trial design

This was a single center, open-label study in which the absolute bioavailability of imatinib (Figure 1a) was determined at steady state by concomitant administration of an intravenous microdose of stable isotopically labeled imatinib-d8 (Figure 1b). Figure 2 provides a schematic overview of the study design. On day 1, patients received a single intravenous microdose of imatinib-d8, next to the standard treatment of imatinib 400 mg once daily (Glivec®). After intake of imatinib at approximately 08:30 a.m., a 100 μg imatinib-d8 microdose was administered intravenously as a bolus injection at the estimated maximum plasma concentration (*t*_max_) of oral imatinib (2.5 h post oral dose). Oral imatinib intake was not interrupted for the duration of the study. The study (Netherlands Trial Register, NTR7642, www.nederlandstrialregister.nl) was approved by both the Medical Ethics Committee of The Netherlands Cancer Institute, Amsterdam, The Netherlands, as well as the competent authority (Centrale Commissie Mensgebonden Onderzoek, CCMO). The study was conducted in accordance with the Declaration of Helsinki. All participants provided written informed consent prior to study assessments.

### Patients

Patients ≥ 18 years of age, treated with imatinib 400 mg once daily in the morning for at least 7 days (steady state plasma concentrations), were included. Subjects needed to have acceptable organ function, as evidenced by laboratory data: aspartate aminotransferase (ASAT) and alanine aminotransferase (ALAT) < 5× the upper limit of normal (ULN), total serum bilirubin ≤ 2× ULN, and renal function as defined by glomerular filtration rate (GFR MDRD) > 40 mL/min/1.73m^2^. Subjects who received treatment with inhibitors or inducers of CYP3A4 were excluded.

### Treatment

Patients received 400 mg imatinib (Glivec®) tablets once daily in the morning as part of routine clinical care. According to the drug label, imatinib was ingested concomitant with a meal. [11] Meals were not standardized. The reference drug imatinib-d8 (Toronto Research Chemicals, ON, Canada) was formulated in the hospital pharmacy of The Netherlands Cancer Institute and was supplied as a 0.1 mg/mL in NaCl 0.9% solution for intravenous injection.

### Sample collection, processing, and analysis

From day 1 to day 3, pharmacokinetic sampling was performed. Blood samples were collected at predose, 0.5, 1, 1.5, 2, 2.5 (pre intravenous microdose), 3, 3.5, 4, 4.5, 5, 6, 8, 12, 24 (pre day 2 oral dose), and 48 h (pre day 3 oral dose), after oral imatinib intake.

Peripheral blood for quantification of imatinib and imatinib-d8 was drawn in 4-mL K_2_ EDTA tubes and centrifuged directly after collection (1500 g, 10 min, 4 °C). Plasma was stored at − 80 °C until analysis. A validated LC-MS/MS assay was used for the simultaneous quantification of imatinib and imatinib-d8. [12] Routine sample analysis acceptance criteria for bioanalytical data according to FDA and EMA guidelines [13, 14] were applied and results were reported using the Analyst 1.6.2. software (Sciex, Framingham, MA, USA).

### Pharmacokinetic analysis and absolute bioavailability calculation

Imatinib and imatinib-d8 plasma concentrations were used to determine the maximum observed plasma concentration (*C*_max_), time to reach maximum plasma concentration (*T*_max_), area under the plasma concentration-time curve from time zero to 24 h (AUC_0–24h_) for imatinib, and from time zero to infinity (AUC_0-inf_) for imatinib-d8, the terminal phase half-life (*t*½) and the elimination rate constant from the central compartment (*k*_e_), the volume of distribution (*V*_d_), and total plasma clearance (CL). Parameters were calculated using plasma concentration-time curves obtained from 0 to 24 h for imatinib, and from 0 to 48 h for imatinib-d8. Non-compartmental analysis was performed using R version 3.0.1. [15]

As the exposure at steady state plasma pharmacokinetics during the dose interval is equivalent to the exposure from zero to infinity following a single administration [16], the AUC_0–24h_ for imatinib and the AUC_0-inf_ for imatinib-d8 could be used to calculate the absolute bioavailability without dose interruptions for the patients.

The absolute bioavailability (*F*) of oral imatinib was calculated as the ratio of dose-normalized exposures of the oral (po) imatinib and intravenous (iv) imatinib-d8 gift expressed as a percentage using the following formula:

1 $$ F\left(\%\right)=\frac{{\left[\mathrm{AU}{\mathrm{C}}_{0-24}\right]}_{\mathrm{po}}/\mathrm{Dos}{\mathrm{e}}_{\mathrm{po}}}{{\left[\mathrm{AU}{\mathrm{C}}_{0-\operatorname{inf}}\right]}_{\mathrm{iv}}/\mathrm{Dos}{\mathrm{e}}_{\mathrm{iv}}}\times 100 $$

## Results

A total of six patients have been included, with a median age of 65 years (range 52–72). Of these patients, 50% received adjuvant imatinib treatment for GIST and 50% were treated in the metastatic setting. An overview of patient baseline characteristics can be found in Table 1.

**Table 1:** Patient baseline characteristics

Characteristic	Patients
Age, years	65 (52–72)
Gender, male	4 (67%)
Tumor type	
GIST	6 (100%)
Treatment setting	
Adjuvant	3 (50%)
Metastatic	3 (50%)
Previous surgery type	
Wedge partial resection of the stomach	3 (50%)
Partial small bowel resection	1 (17%)
Multiple resections*	2 (33%)
Time on imatinib treatment (in years)	3.2 (0.3–13.0)
Albumin (in g/L)	44 (42–47)
eGFR** (in mL/min)	69 (58–84)

Data are expressed as no. (%) or median (range), as appropriate

*eGFR* estimated glomerular filtration rate, *GIST* gastrointestinal stromal tumor

^*^One patient with wedge partial resection of the stomach and partial colon resection, and one patient with wedge partial resection of the stomach, splenectomy, and partial pancreas resection

^**^eGFR was calculated using the MDRD-4 formula

**Fig. 3:**
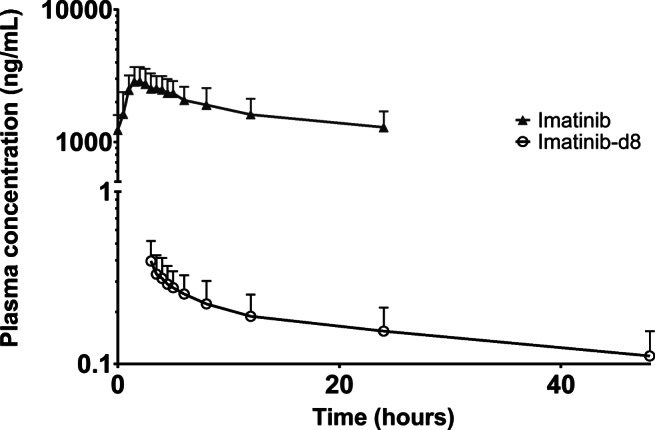
Plasma concentration-time curves of imatinib and imatinib-d8 (mean ± SD) following oral administration of 400 mg imatinib dose at *t* = 0 h and intravenous administration of a 100 μg imatinib-d8 microdose at *t* = 2.5 h in patients (*n* = 6) displaying steady state imatinib plasma pharmacokinetics

All included patients were evaluable for pharmacokinetic analysis. Mean plasma concentration-time curves of imatinib and imatinib-d8 can be found in Fig. 3. A summary of imatinib and imatinib-d8 pharmacokinetic parameters can be found in Table 2.

**Table 2:** Summary of imatinib and imatinib-d8 steady state pharmacokinetic parameters following concomitant administration of an oral imatinib dose (400 mg) and an intravenous imatinib-d8 microdose (100 μg) in cancer patients (*n* = 6)

Parameter		Imatinib	Imatinib-d8
*C*_max_ (μg/mL)	Mean	2.9	0.00051
	CV (%)	27.4	23.1
*C*_min, 0 h_ (μg/mL)	Mean	1.2	N/A
	CV (%)	27.4	N/A
*T*_max_ (h)	Median	2	N/A
	Range	1.5–2	N/A
AUC_0–24_ (μg h/mL)	Mean	42.6	N/A
	CV (%)	30.2	N/A
AUC_0-inf_ (μg h/mL)	Mean	N/A	0.015
	CV (%)	N/A	43.7
*t*½ (h)	Mean	34.1	45.5
	CV (%)	46.7	37.9
*k*_e_ (h^−1^)	Mean	0.023	0.017
	CV (%)	27.5	28.1
*V*_d_/*F* (L)	Mean	190	N/A
	CV (%)	26.7	N/A
*V*_d_ (L)	Mean	N/A	462
	CV (%)	N/A	28.2
CL/*F* (L/h)	Mean	4.2	N/A
	CV (%)	31.3	N/A
CL (L/h)	Mean	N/A	7.6
	CV (%)	N/A	36.1

*AUC*_*0-inf*_ area under the plasma concentration-time curve from time 0 to infinity; *AUC*_*0–24*_ area under the plasma concentration-time curve from time 0 to 24 h; *CL/F* apparent oral clearance; *CL* apparent total body clearance; *C*_*max*_ maximum observed plasma concentration; *C*_*min*_ minimum observed plasma concentration at *t* = 0 h; *CV* coefficient of variation; *N/A* not applicable; *t*_*max*_ time to reach maximum observed plasma concentration; *t*_*½*_ terminal half-life; *V*_*d*_*/F* apparent volume of distribution after oral administration; *V*_*d*_ apparent volume of distribution

The absorption of imatinib after oral administration of tablets was rapid, with a median *t*_max_ of 2 h. The *C*_max_ of oral imatinib at steady state was 2.9 ± 0.8 μg/mL. The mean AUC_0–24_ for oral imatinib was 42.6 ± 12.9 μg h/mL, and the mean AUC_0-inf_ for imatinib-d8 was 0.015 ± 0.007 μg h/mL. The AUC_0-inf_ for imatinib-d8 normalized to a 400-mg imatinib dose was 60.5 ± 26.4 μg h/mL. Individual plasma concentration-time curves demonstrated up to two secondary peaks after the *C*_max_, with different profiles for oral imatinib and intravenous imatinib-d8 (Supplementary Fig. 1). The ratios between the curves for oral imatinib and intravenous imatinib-d8 remained constant during the elimination phase, with a dose-normalized imatinib:imatinib-d8 ratio in plasma of 2.00 at *t* = 6 h and of 2.04 at *t* = 24 h. The *t*½ and clearance of imatinib-d8 were 45.5 h and 7.6 L/h, respectively.

The absolute bioavailability (*F*) of oral imatinib at steady state was calculated for each individual subject. Table 3 demonstrates that the median absolute bioavailability of oral imatinib in cancer patients was 76% (range 42–106%).

**Table 3:** Absolute bioavailability of oral imatinib at steady state plasma pharmacokinetics (n = 6)

	Imatinib tablet (400 mg)	Intravenous imatinib-d8 (100 μg)
AUC_0–24_ (μg h/mL)(CV%)	42.6 (30.2)	N/A
AUC_0-inf_ (μg h/mL) (CV%)	N/A	0.015 (43.7)
Dose-normalized AUC_0-inf_ (μg h/mL) (CV%)	N/A	60.5 (43.7)
*F*(%) (median, range)	76 (42–106)	–

## Discussion

The current study describes results on the determination of the absolute bioavailability of oral imatinib following concomitant administration of a single intravenous stable isotopically labeled imatinib-d8 microdose.

Technically, the stable isotopically microdose trial design proved successful. For all patients, imatinib and imatinib-d8 concentrations could be simultaneously quantified in all collected plasma samples. The quantification of imatinib-d8 was not biased by high concentrations of unlabeled imatinib present in the same plasma sample. In theory, the use of deuterium as a label for the intravenous microdose may result in a kinetic isotope effect (KIE), caused by increased bond strength of the carbon-deuterium bond, as compared with the carbon-hydrogen bond. The KIE may result in altered pharmacokinetics (e.g., altered metabolism) of the deuterium labeled drug, with an incorrect calculation of the absolute bioavailability as a result. [6] As the deuterium labels in the imatinib-d8 structure were not located at metabolic hot spots in the imatinib molecule [17], the KIE was assumed to be negligible. As seen in Fig. 3, the curves for oral and intravenous imatinib demonstrate a parallel decline during the terminal elimination phase, with a constant mean dose-normalized imatinib:imatinib-d8 ratio in plasma of around 2.00, confirming that the KIE for the imatinib-d8 molecule was indeed negligible. The curves presented here demonstrate the validity of using the deuterium labeled imatinib-d8 drug molecule for intravenous microdose administration.

The median absolute bioavailability was calculated to be 76% which was less than the 98% (87–111% (90% confidence interval)) reported using a traditional two-period crossover design in healthy volunteers. [18] There might be different reasons for the lower absolute bioavailability found in this study as compared with the study in healthy volunteers. In the previous absolute bioavailability trial, healthy volunteers demonstrated considerable inter-subject variation in the absolute bioavailability of imatinib in twelve treated subjects. [1] The reasons for the high variability may be attributed to inter-subject variations in the activity of cytochrome P450 isoenzyme 3A4 (CYP3A4), a major enzyme in the biotransformation of imatinib. [1] It could be that the lower bioavailability found in our study may solely be a result of this interpatient variability, as both studies demonstrate a relatively large inter-subject variability in small study populations (6 and 12 subjects included for each trial, respectively). An alternative theory may be that the absolute bioavailability actually differs between healthy volunteers and GIST patients. If so, there might be a change present at baseline, or a change developed during prolonged treatment with imatinib. In theory, GIST disease status may negatively influence the absorption of drug into the systemic circulation, resulting in a lower absolute bioavailability at baseline. In a previous study, patients with a prior major gastrectomy had a significantly lower *C*_min,_ while other types of surgery were not associated with decreased pharmacokinetic exposure. [19] However, in another observational study, type of surgery and extent of resection were not predictive of low imatinib concentrations. [20] Our study patient population consisted of patients without prior major gastrectomy (Table 1), and results were therefore not likely to be influenced by prior surgery.

Another explanation for the lower bioavailability might be a change developed during prolonged imatinib treatment. Imatinib pharmacokinetic parameters have been described to change from early to later treatment phase, with a trend towards increased imatinib clearance after long-term exposure [21, 22], although this finding could not be reproduced in other studies. [20, 23] In our study population, all patients were on imatinib treatment for several months or years (median 3.2 years, range 0.3–13.0 years), and the clearance was similar to the clearance observed during the first month of treatment as described by Judson et al. (7.6 L/h vs. 9.2 L/h). [21] Since pharmacokinetic exposure to imatinib has been related to treatment efficacy [24], therapeutic drug monitoring has been implemented in our hospital. Therefore, in case of an increased clearance and thus a lower pharmacokinetic exposure, dose escalations have probably been performed. These patients were not eligible for inclusion in this trial, which might explain the absence of an observed increase in drug clearance as a result of selection bias. Furthermore, if a change in clearance was found, this would not have explained the lower value for absolute bioavailability, as the oral and intravenous dose are co-administered during a single-dose event, eliminating inter-dose variability.

In a prospective pharmacokinetic trial on imatinib plasma concentrations in GIST patients, a reduced exposure of approximately 30% to imatinib was observed after long-term treatment (> 90 days), most likely due to reduced drug absorption over time. [25] This reduced exposure may potentially be a result of the lower absolute bioavailability that we observed in our trial. Although different theories for this reduced absorption and/or bioavailability do exist (e.g., changed activity or expression of drug transporters involved in active transport, upregulation of CYP3A4) [25], none has been confirmed to date.

Finally, the lower bioavailability found in our study could potentially be explained by the fact that patients ingested imatinib concomitant with food (according to the label), while the previous absolute bioavailability study has been performed under fasted conditions. Although a previous food-effect study concluded that food did not affect imatinib pharmacokinetics to a clinically relevant extent, *C*_max_ and AUC_0–24h_ decreased 15% and 9%, respectively, after concomitant intake with a high-fat meal compared with the fasted state. [26]

Interestingly, the individual plasma concentration-time curves demonstrated up to two secondary peaks after the *C*_max_, with different profiles for oral imatinib and intravenous imatinib-d8 (Supplementary Fig. 1). Previous research on imatinib has not demonstrated enterohepatic cycling of imatinib. Another explanation for these peaks might be bile secretion triggered by food intake, resulting in acceleration of drug solubility in the gastrointestinal lumen, although food has been described to have no relevant impact on the rate or extent of bioavailability. [27]

By using the stable isotopically labeled microdose trial design, a number of dose events and collected plasma samples were reduced by half, as compared with the previously performed absolute bioavailability trial using a conventional crossover design. [18] This reduction may aid to perform this trial in patients in the future, as it offers the possibility to be combined with a phase I/II trial in patients without adding a separate intravenous dose event. The microdose trial design using a stable isotopically labeled drug will only mildly increase patient burden by adding a single intravenous microdose administration to the study procedures. This minor adjustment may result in increased and more relevant knowledge on the pharmacokinetics of a novel drug product in an early stage of clinical drug development.

## Conclusion

The absolute bioavailability of oral imatinib in cancer patients during steady state pharmacokinetics was successfully determined using a stable isotopically labeled microdose trial. This study demonstrates the potential to use a stable isotopically labeled microdose in combination with LC-MS/MS for the assessment of absolute bioavailability. In addition, the potential added value of performing an absolute bioavailability study in the intended patient population for clinical use during steady state pharmacokinetics was demonstrated by comparing the results obtained with a previously performed absolute bioavailability trial in healthy volunteers.

## Electronic supplementary material

ESM 1 (DOCX 506 kb)
