# Supplementary material for: Determination of the absolute bioavailability of oral imatinib using a stable isotopically labeled intravenous imatinib-d8 microdose
Source: Eur J Clin Pharmacol. 2020 May 19;76(8):1075–82. doi: 10.1007/s00228-020-02888-y (PMC7351863; doi:10.1007/s00228-020-02888-y)
Supplement: Supplementary file 1 — (DOCX 506 kb) [file 228_2020_2888_MOESM1_ESM.docx]

**Determination of the absolute bioavailability of oral imatinib using a stable isotopically labeled intravenous imatinib-d8 microdose**

Jeroen Roosendaal^1^, Stefanie L. Groenland^2^, Hilde Rosing^1^, Luc Lucas^1^, Nikkie Venekamp^1^, Bastiaan Nuijen^1^, Alwin D.R. Huitema^1,3^, Jos H. Beijnen^1,4^, Neeltje Steeghs^2^

^1^ Department of Pharmacy & Pharmacology, Netherlands Cancer Institute – Antoni van Leeuwenhoek, Amsterdam, The Netherlands

^2^ Department of Medical Oncology and Clinical Pharmacology, The Netherlands Cancer Institute – Antoni van Leeuwenhoek, Amsterdam, The Netherlands.

^3^ Department of Clinical Pharmacy, University Medical Center, Utrecht University, Utrecht, The Netherlands.

^4^ Division of Pharmacoepidemiology and Clinical Pharmacology, Faculty of Science, Utrecht Institute for Pharmaceutical Sciences, Utrecht University, Utrecht, The Netherlands.

Correspondence: Jeroen Roosendaal, PharmD, Department of Pharmacy & Pharmacology, Netherlands Cancer Institute, Plesmanlaan 121, 1066 CX, Amsterdam, The Netherlands. Phone: +31 20 512 7928; E-mail: j.roosendaal@nki.nl

Journal: European Journal of Clinical Pharmacology (Original Research)

Word count abstract: 250

Word count: 2651

Figures: 3

Tables: 3

Supplementary figures: 1

References: 24

**ABSTRACT**

**Purpose**The aim of this study was to ascertain whether the absolute bioavailability of oral imatinib (Glivec^®^) during steady state plasma pharmacokinetics in cancer patients could be determined through a concomitant intravenous administration of a single 100 µg microdose of deuterium labeled imatinib (imatinib-d8). Secondly, the usefulness of liquid chromatography – tandem mass spectrometry (LC-MS/MS) was investigated for simultaneous analysis of orally and intravenously administered imatinib.

**Methods**Included patients were on a stable daily dose of 400 mg oral imatinib prior to study participation. On day 1, patients received a 100 µg intravenous imatinib-d8 microdose 2.5 h after intake of the oral dose. Plasma samples were collected for 48 h. Imatinib and imatinib-d8 concentrations were simultaneously quantified using a validated LC-MS/MS assay. The absolute bioavailability was calculated by comparing the dose-normalized exposure to unlabeled and stable isotopically labeled imatinib in plasma.

**Results**A total of six patients were enrolled. All patients had a history of gastro-intestinal stromal tumors (GIST). The median absolute bioavailability of oral imatinib at steady state was 76% (range: 44-106%). Imatinib and imatinib-d8 plasma concentrations were quantified in all collected plasma samples, with no samples below the limit of quantification for imatinib-d8.

**Conclusion**The absolute bioavailability of imatinib was successfully estimated at steady state plasma pharmacokinetics using the stable isotopically labeled microdose trial design. This study exhibits the use of a stable isotopically labeled intravenous microdose to determine the absolute bioavailability of an oral anticancer agent in patients with LC-MS/MS as the analytical tool.

**KEYWORDS**

Imatinib, microdosing, absolute bioavailability, stable isotope labeled, steady state

**INTRODUCTION**

The last decade has shown an increasing number of anticancer drugs that are administered orally. [1–3] This so called ‘intravenous to oral switch’ in oncology has resulted in an increased attention on the investigation of the absolute bioavailability during clinical drug development. Determining the absolute oral bioavailability of a new drug candidate facilitates the identification of potential developmental challenges such as absorption and first pass metabolism during the clinical development of a drug. Hence, the assessment of the absolute bioavailability is also crucial for the development of optimized oral formulations. As a result, data on the absolute bioavailability of novel oral drugs is now increasingly requested by the FDA and EMA, [4,5]

The conventional way to assess the absolute oral bioavailability is by using a two-period crossover study design, where an intravenous dose and an oral dose are administered to a study subject with a washout period in between. The absolute bioavailability is then calculated by dividing the plasma exposure after oral administration by the plasma exposure after intravenous administration. A limitation of this design is that for drugs that are poorly soluble in aqueous media it might be impossible to develop an intravenous formulation at therapeutic strength. In addition, it assumes linear pharmacokinetics and constant clearance between the oral and intravenous dose event, which might not always be the case for drugs demonstrating a high intra-patient variability. This may result in a systemic error in the determination of the absolute bioavailability. [6]

A study design of co-administering an intravenous isotopically labeled microdose (≤100 μg, less than 1/100^th^ of the therapeutic dose) with a therapeutic oral dose provides a solution to these problems. Because only a small amount of drug needs to be dissolved in an intravenous formulation, drug solubility issues can be circumvented. In addition, according to the current regulatory guidelines, clinical intravenous microdose studies could be carried out without additional toxicity investigations, saving costs and time associated with intravenous drug development. [7] Furthermore, because the intravenous microdose is administered during the same dose event as the oral therapeutic dose, the study duration is shortened and intra-occasion variability is not an issue, resulting in a more accurate determination of the absolute bioavailability and increased patients convenience.

Absolute bioavailability microdose trials can be performed using either radiolabeled or stable isotopically labeled drug processed into an intravenous formulation. In recent years, accelerator mass spectrometry (AMS) to measure a radiolabeled microdose has been utilized to support several clinical absolute bioavailability studies. [8] A drawback of AMS is that sample analysis is labour- and time intensive, expensive, and that AMS is only available in a limited number of places dedicated to biomedical research worldwide. [9] An alternative analytical approach for conducting microdose studies is using liquid chromatography coupled to tandem mass spectrometry (LC-MS/MS) to quantitate both the intravenous and the oral drug. Because both labeled and unlabeled drug can be measured simultaneously with LC-MS/MS, it is an elegant and cost effective alternative to AMS. [9,10]

For the group of tyrosine kinase inhibitors, an important class of novel oral anticancer agents, it has been demonstrated that for many drugs registered in the past years the absolute bioavailability has not been assessed at the time of drug licensing. [3] One reason for this might be that poor drug solubility hampers the development of an aqueous intravenous dose at therapeutic strength, making it almost impossible to use the conventional crossover trial design. In this trial we used imatinib, a tyrosine kinase inhibitor used for the treatment of chronic myeloid leukemia (CML) and gastro-intestinal stromal tumors (GIST), to demonstrate the potential of using a stable isotopically labeled 100 µg microdose in combination with LC-MS/MS to assess the absolute bioavailability.

The objective of this study was to ascertain whether the absolute bioavailability of oral imatinib (Glivec^®^) during steady state plasma pharmacokinetics in cancer patients could be determined through a concomitant intravenous administration of a single 100 µg microdose of deuterium labeled imatinib (imatinib-d8). Secondly, the usefulness of liquid chromatography – tandem mass spectrometry (LC-MS/MS) is investigated for simultaneous analysis of orally and intravenously administered imatinib.

**MATERIALS AND METHODS**

*Study design and sample collection*

This was a single center, open-label study in which the absolute bioavailability of imatinib was determined at steady state by concomitant administration of an intravenous microdose of stable isotopically labeled imatinib-d8. Figure 1 provides a schematic overview of the study design. On day 1, patients received a single intravenous microdose of imatinib-d8, next to the standard treatment of imatinib 400 mg once daily (Glivec®). After intake of imatinib at approximately 08:30 a.m., a 100 µg imatinib-d8 microdose was administered intravenously as a bolus injection at the estimated maximum plasma concentration (t_max_) of oral imatinib (2.5 hours post oral dose). Oral imatinib intake was not interrupted for the duration of the study. The study (Netherlands Trial Register, NTR7642, www.nederlandstrialregister.nl) was approved by both the Medical Ethics Committee of The Netherlands Cancer Institute, Amsterdam, The Netherlands, as well as the competent authority (Centrale Commissie Mensgebonden Onderzoek, CCMO). The study was conducted in accordance with the Declaration of Helsinki. All participants provided written informed consent prior to study assessments.

*Patients*

Patients ≥ 18 years of age, treated with imatinib 400 mg once daily in the morning for at least 7 days (steady state plasma concentrations), were included. Subjects needed to have acceptable organ function, as evidenced by laboratory data: aspartate aminotransferase (ASAT) and alanine aminotransferase (ALAT) <5x the upper limit of normal (ULN), total serum bilirubin ≤2x ULN, renal function as defined by glomerular filtration rate (GFR MDRD) >40 mL/min/1.73m^2^. Subjects who received treatment with inhibitors or inducers of CYP3A4 were excluded.

*Treatment*

Patients received 400 mg imatinib (Glivec^®^) tablets once daily in the morning as part of routine clinical care. According to the drug label, imatinib was ingested concomitant with a meal. [11] Meals were not standardized. The reference drug imatinib-d8 (Toronto Research Chemicals, ON, Canada) was formulated in the hospital pharmacy of The Netherlands Cancer Institute and was supplied as a 0.1 mg/mL in NaCl 0.9% solution for intravenous injection.

*Sample collection, processing and analysis*

From day 1 to day 3, pharmacokinetic sampling was performed. Blood samples were collected at predose, 0.5, 1, 1.5, 2, 2.5 (pre intravenous microdose), 3, 3.5, 4, 4.5, 5, 6, 8, 12, 24 (pre day 2 oral dose) and 48 h (pre day 3 oral dose), after oral imatinib intake.

Peripheral blood for quantification of imatinib and imatinib-d8 was drawn in 4mL K_2_ EDTA tubes and centrifuged directly after collection (1500 g, 10 min, 4°C). Plasma was stored at -80 °C until analysis. A validated LC-MS/MS assay was used for the simultaneous quantification of imatinib and imatinib-d8. [12] Routine sample analysis acceptance criteria for bioanalytical data according to FDA and EMA guidelines [13,14] were applied and results were reported using Analyst 1.6.2. software (Sciex, Framingham, MA, USA).

*Pharmacokinetic analysis and absolute bioavailability calculation*

Imatinib and imatinib-d8 plasma concentrations were used to determine the maximum observed plasma concentration (C_max_), time to reach maximum plasma concentration (T_max_), area under the plasma concentration-time curve from time zero to 24 h (AUC_0-24h_) for imatinib, and from time zero to infinity (AUC_0-inf_) for imatinib-d8, the terminal phase half-life (t½) and the elimination rate constant from the central compartment (k_e_), the volume of distribution (V_d_), and total plasma clearance (CL). Parameters were calculated using plasma concentration-time curves obtained from 0 – 24 h for imatinib, and from 0 – 48 h for imatinib-d8. Non-compartmental analysis was performed using R version 3.0.1. [15]

As the exposure at steady state plasma pharmacokinetics during the dose interval is equivalent to the exposure from zero to infinity following a single administration [16], the AUC_0-24h_ for imatinib and the AUC_0-inf_ for imatinib-d8 could be used to calculate the absolute bioavailability without dose interruptions for the patients.

The absolute bioavailability (F) of oral imatinib was calculated as the ratio of dose-normalized exposures of the oral (po) imatinib and intravenous (iv) imatinib-d8 gift expressed as a percentage using the following formula:

1. $F\left( \% \right)=\frac{\left[ AUC_{0-24} \right]_{po}/Dose_{po}}{\left[ AUC_{0-inf} \right]_{iv}/Dose_{iv}} x 100$

**RESULTS**A total of six patients have been included, with a median age of 65 years (range 52-72). Of these patients, 50% received adjuvant imatinib treatment for GIST and 50% was treated in the metastatic setting. An overview of patient baseline characteristics can be found in Table 1.

All included patients were evaluable for pharmacokinetic analysis. Mean plasma concentration-time curves of imatinib and imatinib-d8 can be found in Figure 3. A summary of imatinib and imatinib-d8 pharmacokinetic parameters can be found in Table 2.

The absorption of imatinib after oral administration of tablets was rapid, with a median t_max_ of 2 hours. The C_max_ of oral imatinib at steady state was 2.9 ± 0.8 µg/mL. The mean AUC_0-24_ for oral imatinib was 42.6 ± 12.9 µg·h/mL, and the mean AUC_0-inf_ for imatinib-d8 was 0.015 ± 0.007 µg·h/mL. The AUC_0-inf_ for imatinib-d8 normalized to a 400 mg imatinib dose was 60.5 ± 26.4 µg·h/mL. Individual plasma concentration-time curves demonstrated up to two secondary peaks after the C_max_, with different profiles for oral imatinib and intravenous imatinib-d8 (Supplementary Figure 1). The ratios between the curves for oral imatinib and intravenous imatinib-d8 remained constant during the elimination phase, with a dose-normalized imatinib:imatinib-d8 ratio in plasma of 2.00 at t=6h and of 2.04 at t=24h. The t½ and clearance of imatinib-d8 were 45.5 h and 7.6 L/h, respectively.

The absolute bioavailability (F) of oral imatinib at steady state was calculated for each individual subject. Table 3 demonstrates that the median absolute bioavailability of oral imatinib in cancer patients was 76% (range 42% - 106%).

**DISCUSSION**

The current study describes results on the determination of the absolute bioavailability of oral imatinib following concomitant administration of a single intravenous stable isotopically labeled imatinib-d8 microdose.

Technically, the stable isotopically microdose trial design proved successful. For all patients, imatinib and imatinib-d8 concentrations could be simultaneously quantified in all collected plasma samples. The quantification of imatinib-d8 was not biased by high concentrations of unlabeled imatinib present in the same plasma sample. In theory, the use of deuterium as a label for the intravenous microdose may result in a kinetic isotope effect (KIE), caused by increased bond strength of the carbon-deuterium bond, as compared to the carbon-hydrogen bond. The KIE may result in altered pharmacokinetics (eg altered metabolism) of the deuterium labeled drug, with an incorrect calculation of the absolute bioavailability as a result. [6] As the deuterium labels in the imatinib-d8 structure were not located at metabolic hot spots in the imatinib molecule [17], the KIE was assumed to be negligible. As seen in Figure 3, the curves for oral and intravenous imatinib demonstrate a parallel decline during the terminal elimination phase, with a constant mean dose-normalized imatinib:imatinib-d8 ratio in plasma of around 2.00, confirming that the KIE for the imatinib-d8 molecule was indeed negligible. The curves presented here demonstrate the validity of using the deuterium labeled imatinib-d8 drug molecule for intravenous microdose administration.

The median absolute bioavailability was calculated to be 76% which was less than the 98% (87% - 111% [90% confidence interval]) reported using a traditional two period crossover design in healthy volunteers. [18] There might be different reasons for the lower absolute bioavailability found in this study as compared to the study in healthy volunteers. In the previous absolute bioavailability trial, healthy volunteers demonstrated considerable inter subject variation in the absolute bioavailability of imatinib in twelve treated subjects. [1] The reasons for the high variability may be attributed to inter subject variations in the activity of cytochrome P450 isoenzyme 3A4 (CYP3A4), a major enzyme in the biotransformation of imatinib. [1] It could be that the lower bioavailability found in our study may solely be a result of this interpatient variability, as both studies demonstrate a relatively large inter-subject variability in small study populations (6 and 12 subjects included for each trial, respectively). An alternative theory may be that the absolute bioavailability actually differs between healthy volunteers and GIST patients. If so, there might be a change present at baseline, or a change developed during prolonged treatment with imatinib. In theory, GIST disease status may negatively influence the absorption of drug into the systemic circulation, resulting in a lower absolute bioavailability at baseline. In a previous study, patients with a prior major gastrectomy had a significantly lower C_min,_ while other types of surgery were not associated with decreased pharmacokinetic exposure. [19] However, in another observational study, type of surgery and extent of resection were not predictive of low imatinib concentrations. [20] Our study patient population consisted of patients without prior major gastrectomy (Table 1), and results were therefore not likely to be influenced by prior surgery.

Another explanation for the lower bioavailability might be a change developed during prolonged imatinib treatment. Imatinib pharmacokinetic parameters have been described to change from early to later treatment phase, with a trend towards increased imatinib clearance after long-term exposure [21,22], although this finding could not be reproduced in other studies. [20,23] In our study population, all patients were on imatinib treatment for several months or years (median 3.2 years, range 0.3 – 13.0 years), and the clearance was similar to the clearance observed during the first month of treatment as described by Judson et al (7.6 L/h vs 9.2 L/h). [21] Since pharmacokinetic exposure to imatinib has been related to treatment efficacy [24], therapeutic drug monitoring has been implemented in our hospital. Therefore, in case of an increased clearance and thus a lower pharmacokinetic exposure, dose escalations have probably been performed. These patients were not eligible for inclusion in this trial, which might explain the absence of an observed increase in drug clearance as a result of selection bias. Furthermore, if a change in clearance was found, this would not have explained the lower value for absolute bioavailability, as the oral and intravenous dose are co-administered during a single dose event, eliminating inter dose variability.

In a prospective pharmacokinetic trial on imatinib plasma concentrations in GIST patients, a reduced exposure of approximately 30% to imatinib was observed after long-term treatment (>90 days), most likely due to reduced drug absorption over time. [25] This reduced exposure may potentially be a result of the lower absolute bioavailability that we observed in our trial. Although different theories for this reduced absorption and/or bioavailability do exist (e.g. changed activity or expression of drug transporters involved in active transport, upregulation of CYP3A4) [25], none have been confirmed to date.

Finally, the lower bioavailability found in our study could potentially be explained by the fact that patients ingested imatinib concomitant with food (according to the label), while the previous absolute bioavailability study has been performed under fasted conditions. Although a previous food-effect study concluded that food did not affect imatinib pharmacokinetics to a clinically relevant extent, C_max_ and AUC_0-24h_ decreased 15% and 9%, respectively, after concomitant intake with a high-fat meal compared to the fasted state. [26]

Interestingly, the individual plasma concentration-time curves demonstrated up to two secondary peaks after the C_max_, with different profiles for oral imatinib and intravenous imatinib-d8 (Supplementary Figure 1). Previous research on imatinib has not demonstrated enterohepatic cycling of imatinib. Another explanation for these peaks might be bile secretion triggered by food intake, resulting in acceleration of drug solubility in the gastrointestinal lumen, although food has been described to have no relevant impact on the rate or extent of bioavailability. [24]

By using the stable isotopically labeled microdose trial design, the number of dose events and collected plasma samples were reduced by half, as compared to the previously performed absolute bioavailability trial using a conventional crossover design. [18] This reduction may aid to perform this trial in patients in the future, as it offers the possibility to be combined with a phase I/II trial in patients without adding a separate intravenous dose event. The microdose trial design using a stable isotopically labeled drug will only mildly increase patient burden by adding a single intravenous microdose administration to the study procedures. This minor adjustment may result in increased and more relevant knowledge on the pharmacokinetics of a novel drug product in an early stage of clinical drug development.

**CONCLUSION**

The absolute bioavailability of oral imatinib in cancer patients during steady state pharmacokinetics was successfully determined using a stable isotopically labeled microdose trial. This study demonstrates the potential to use a stable isotopically labeled microdose in combination with LC-MS/MS for the assessment of absolute bioavailability. In addition, the potential added value of performing an absolute bioavailability study in the intended patient population for clinical use during steady state pharmacokinetics was demonstrated by comparing the results obtained to a previously performed absolute bioavailability trial in healthy volunteers.

**COMPETING INTERESTS**There are no competing interests to declare in relation to this study.

**CONTRIBUTORS**

J.R., H.R., B.N., A.H., J.B. and N.S. were involved in conception and design of the study. J.R., S.G., N.V., A.H., J.B. and N.S. were involved in acquisition, analysis and interpretation of the data. J.R. drafted the manuscript and all other authors critically revised the manuscript. All authors gave final approval for the manuscript to be published. N.S. was the principal investigator.

**REFERENCES**

1. Liu G, Franssen E, Fitch MI, Warner E. Patient preferences for oral versus intravenous palliative chemotherapy. J Clin Oncol. 1997;15:110–5.

2. Benjamin L, Cotté FE, Philippe C, Mercier F, Bachelot T, Vidal-Trécan G. Physicians’ preferences for prescribing oral and intravenous anticancer drugs: A Discrete Choice Experiment. Eur J Cancer. 2012;48:912–20.

3. Herbrink M, Nuijen B, Schellens JHM, Beijnen JH. Variability in bioavailability of small molecular tyrosine kinase inhibitors. Cancer Treat. Rev. 2015;41:412-422

4. European Medicines Agency. Clinical pharmacology and pharmacokinetics : questions and answers [cited 2019 Apr 15]. Available from: https://www.ema.europa.eu/en/human-regulatory/research-development/scientific-guidelines/clinical-pharmacology-pharmacokinetics/clinical-pharmacology-pharmacokinetics-questions-answers

5. U.S. Food and Drug Administration. Bioavailability Studies Submitted in NDAs or INDs — General Considerations Guidance for Industry [cited 2020 Apr 21]. Available from: https://www.fda.gov/media/121311/download

6. Lappin G, Rowland M, Garner RC. The use of isotopes in the determination of absolute bioavailability of drugs in humans. Expert Opin Drug Metab Toxicol. 2006;2:419–27.

7. European Medicines Agency. ICH M3(R2) - Guidance on non-clinical safety studies for the conduct of human clinical trials and marketing authorization for pharmaceuticals. [cited 2019 Apr 15]. Available from: http://www.ich.org/fileadmin/Public_Web_Site/ICH_Products/Guidelines/Multidisciplinary/M3_R2/Step4/M3_R2__Guideline.pdf

8. Xu X, Jiang H, Christopher LJ, Shen JX, Zeng J, Arnold ME. Sensitivity-based analytical approaches to support human absolute bioavailability studies. Bioanalysis. 2014;6:497-504

9. Burt T, John CS, Ruckle JL, Vuong LT. Phase-0/microdosing studies using PET, AMS, and LC-MS/MS: a range of study methodologies and conduct considerations. Accelerating development of novel pharmaceuticals through safe testing in humans–a practical guide. Expert Opin. Drug Deliv. 2017. p. 657–72.

10. Lappin G. Approaches to intravenous clinical pharmacokinetics: Recent developments with isotopic microtracers. J. Clin. Pharmacol. 2016;14:657-672

11. European Medicines Agency. Glivec - Summary of Product Characteristics.[cited 2020 Apr 21]. Available from: https://www.ema.europa.eu/en/documents/product-information/glivec-epar-product-information_en.pdf

12. Roosendaal J, Venekamp N, Lucas L, Rosing H, Beijnen JH. Development and validation of an LC-MS/MS method for the quantification of imatinib and imatinib-d8 in human plasma for the support of an absolute bioavailability microdose trial. Pharmazie. 2020;75:136–41.

13. U.S. Food and Drug Administration. Guidance for Industry: Bioanalytical Method Validation. [cited 2018 Jul 24]. Available from: https://www.fda.gov/downloads/drugs/guidances/ucm070107.Pdf

14. European Medicines Agency. Guideline on bioanalytical method validation. [cited 2018 May 4]. Available from: http://www.ema.europa.eu/ema/index.jsp?curl=pages/includes/document/document_detail.jsp?webContentId=WC500109686&mid=WC0b01ac058009a3dc

15. R Development Core Team, R Core Team. R: A Language and Environment for Statistical Computing. Available from: http://www.r-project.org

16. Rowland M, Tozer TN. Clinical Pharmacokinetics - Concepts and applications. 3rd ed. 1995.

17. Gschwind HP, Pfaar U, Waldmeier F, Zollinger M, Sayer C, Gross G. Metabolism and Disposition of Imatinib Mesylate in Healthy Volunteers Abstract : Drug Metab Dispos. 2005;33:1503–12.

18. Peng B, Dutreix C, Mehring G, Hayes MJ, Ben-Am M, Seiberling M, et al. Absolute Bioavailability of Imatinib (Glivec®) Orally versus Intravenous Infusion. J Clin Pharmacol. 2004;44:158–62.

19. Yoo C, Ryu MH, Kang BW, Yoon SK, Ryoo BY, Chang HM, et al. Cross-sectional study of imatinib plasma trough levels in patients with advanced gastrointestinal stromal tumors: Impact of gastrointestinal resection on exposure to imatinib. J Clin Oncol. 2010;28:1554–9.

20. Farag S, Verheijen RB, Martijn Kerst J, Cats A, Huitema ADR, Steeghs N. Imatinib Pharmacokinetics in a Large Observational Cohort of Gastrointestinal Stromal Tumour Patients. Clin Pharmacokinet. 2017;56:287–92.

21. Judson I, Ma P, Peng B, Verweij J, Racine A, Di Paola ED, et al. Imatinib pharmacokinetics in patients with gastrointestinal stromal tumour: A retrospective population pharmacokinetic study over time. EORTC Soft Tissue and Bone Sarcoma Group. Cancer Chemother Pharmacol. 2005;55:379–86.

22. Bins S, Eechoute K, Kloth JSL, de Man FM, Oosten AW, de Bruijn P, et al. Prospective Analysis in GIST Patients on the Role of Alpha-1 Acid Glycoprotein in Imatinib Exposure. Clin Pharmacokinet. 2017;56:305–10.

23. Yoo C, Ryu MH, Ryoo BY, Beck MY, Chang HM, Lee JL, et al. Changes in imatinib plasma trough level during long-term treatment of patients with advanced gastrointestinal stromal tumors: Correlation between changes in covariates and imatinib exposure. Invest New Drugs. 2012;30:1703–8.

24. Demetri GD, Wang Y, Wehrle E, Racine A, Nikolova Z, Blanke CD, et al. Imatinib plasma levels are correlated with clinical benefit in patients with unresectable/metastatic gastrointestinal stromal tumors. J Clin Oncol. 2009;27:3141–7.

25. Eechoute K, Fransson MN, Reyners AK, De Jong FA, Sparreboom A, Van Der Graaf WTA, et al. A long-term prospective population pharmacokinetic study on imatinib plasma concentrations in GIST patients. Clin Cancer Res. 2012;18:5780-5787

26. U.S. Food and Drug Administration. Gleevec - Clinical Pharmacology and Biopharmaceutics Review. p. 67–70. [cited 2020 Apr 21] Available from: https://www.accessdata.fda.gov/drugsatfda_docs/nda/2001/21-335_Gleevec_biopharmr_P1.pdf

27. Peng B, Lloyd P, Schran H. Clinical pharmacokinetics of imatinib. Clin. Pharmacokinet. 2005;44:879–94.

**FIGURES AND TABLES**

**Figure 1.** Schematic overview of the imatinib absolute bioavailability microdose trial design

**Figure 2.** Molecular structures of imatinib and imatinib-d8

**Figure 3.** Plasma concentration-time profiles of imatinib and imatinib-d8 (mean ±SD) following oral administration of 400 mg imatinib dose at t=0h and intravenous administration of a 100 µg imatinib-d8 microdose at t=2.5h in patients (n=6) displaying steady state imatinib plasma pharmacokinetics

**Table 1.** Patient baseline characteristics

**Table 2.** Summary of imatinib and imatinib-d8 steady state pharmacokinetic parameters following concomitant administration of an oral imatinib dose (400 mg) and an intravenous imatinib-d8 microdose (100 µg) in cancer patients (n=6)

**Table 3.** Absolute bioavailability of oral imatinib at steady state plasma pharmacokinetics (n=6)

**SUPPLEMENTARY FIGURES**

**Supplementary Figure 1.** Plasma concentration-time curves of imatinib and imatinib-d8 following oral (p.o.) administration of 400 mg imatinib dose at t=0h and intravenous (i.v.) administration of a 100 µg imatinib-d8 microdose at t=2.5h in patients (1-6, **A-F**) displaying steady state imatinib plasma pharmacokinetics. **Figure 1.** Schematic overview of the imatinib absolute bioavailability microdose trial design


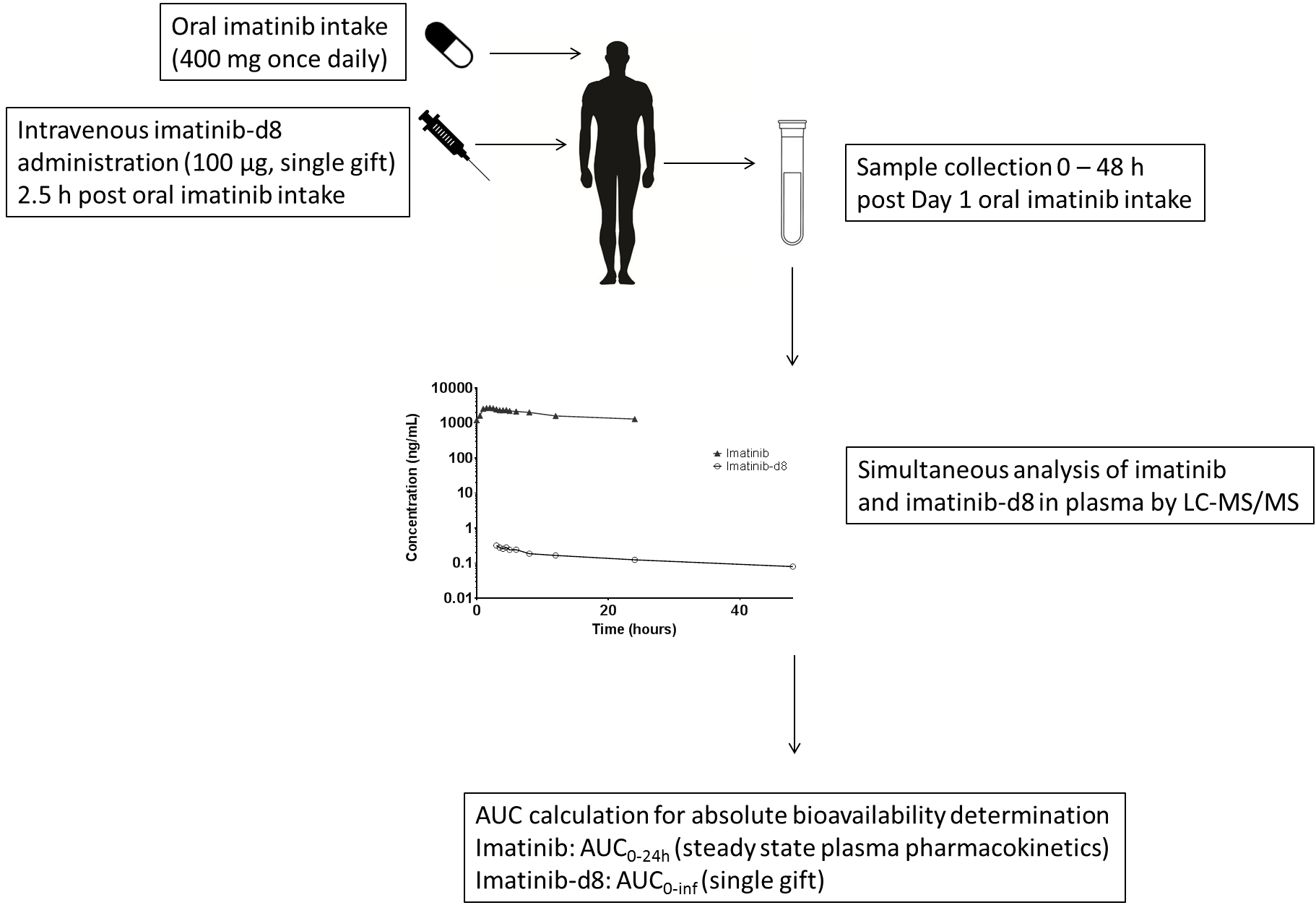


Abbreviations: LC-MS/MS, liquid chromatography coupled to tandem mass spectrometry; AUC_0-24h_, area under the plasma-time curve up to 24 hours, AUC_0-inf_, area under the plasma-time curve extrapolated to infinity **Figure 2.** Molecular structures of **(A)** imatinib and **(B)** imatinib-d8

**A B**

**Figure 3.** Plasma concentration-time curves of imatinib and imatinib-d8 (mean ±SD) following oral administration of 400 mg imatinib dose at t=0h and intravenous administration of a 100 µg imatinib-d8 microdose at t=2.5h in patients (n=6) displaying steady state imatinib plasma pharmacokinetics

**Table 1.** Patient baseline characteristics

| Characteristic | Patients |
| --- | --- |
| Age, years | 65 (52 – 72) |
| Gender, male | 4 (67%) |
| Tumor type  GIST | 6 (100%) |
| Treatment setting  Adjuvant  Metastatic | 3 (50%)  3 (50%) |
| Previous surgery type  Wedge partial resection of the stomach  Partial small bowel resection  Multiple resections* | 3 (50%)  1 (17%)  2 (33%) |
| Time on imatinib treatment (in years) | 3.2 (0.3 – 13.0) |
| Albumin (in g/L) | 44 (42 – 47) |
| eGFR** (in mL/min) | 69 (58 – 84) |

*Data are expressed as no. (%) or median (range), as appropriate.*

** one patient with wedge partial resection of the stomach and partial colon resection, one patient with wedge partial resection of the stomach, splenectomy and partial pancreas resection*

*** eGFR was calculated using the MDRD-4 formula*

*eGFR = estimated glomerular filtration rate, GIST = gastro-intestinal stromal tumour*

**Table 2.** Summary of imatinib and imatinib-d8 steady state pharmacokinetic parameters following concomitant administration of an oral imatinib dose (400 mg) and an intravenous imatinib-d8 microdose (100 µg) in cancer patients (n=6)

| Parameter |  | Imatinib | Imatinib-d8 |
| --- | --- | --- | --- |
| C_max_ (µg/mL) | Mean | 2.9 | 0.00051 |
|  | CV (%) | 27.4 | 23.1 |
| C_min, 0h_ (µg/mL) | Mean | 1.2 | N/A |
|  | CV (%) | 27.4 | N/A |
| T_max_ (h) | Median | 2 | N/A |
|  | Range | 1.5 - 2 | N/A |
| AUC_0-24_ (µg·h/mL) | Mean | 42.6 | N/A |
|  | CV (%) | 30.2 | N/A |
| AUC_0-inf_ (µg·h/mL) | Mean | N/A | 0.015 |
|  | CV (%) | N/A | 43.7 |
| t½ (h) | Mean | 34.1 | 45.5 |
|  | CV (%) | 46.7 | 37.9 |
| k_e_ (h^-1^) | Mean | 0.023 | 0.017 |
|  | CV (%) | 27.5 | 28.1 |
| V_d_/F (L) | Mean | 190 | N/A |
|  | CV (%) | 26.7 | N/A |
| V_d_ (L) | Mean | N/A | 462 |
|  | CV (%) | N/A | 28.2 |
| CL/F (L/h) | Mean | 4.2 | N/A |
|  | CV (%) | 31.3 | N/A |
| CL (L/h) | Mean | N/A | 7.6 |
|  | CV (%) | N/A | 36.1 |

Abbreviations: AUC_0-inf_, Area under the plasma concentration-time curve from time 0 to infinity; AUC_0-24_, Area under the plasma concentration-time curve from time 0 to 24 h; CL/F, Apparent oral clearance; CL, Apparent total body clearance; C_max_, maximum observed plasma concentration; C_min_, minimum observed plasma concentration at t=0 h; CV, Coefficient of variation; N/A, Not applicable; t_max_, time to reach maximum observed plasma concentration; t_½_, terminal half-life; V_d_/F, Apparent volume of distribution after oral administration; V_d_, Apparent volume of distribution. **Table 3.** Absolute bioavailability of oral imatinib at steady state plasma pharmacokinetics (n=6)

|  | **Imatinib tablet**  **(400 mg)** | **Intravenous imatinib-d8**  **(100 µg)** |
| --- | --- | --- |
| AUC_0-24_ (µg·h/mL)(CV%) | 42.6 (30.2) | N/A |
| AUC_0-inf_ (µg·h/mL) (CV%) | N/A | 0.015 (43.7) |
| Dose normalized AUC_0-inf_ (µg·h/mL) (CV%) | N/A | 60.5 (43.7) |
| **F(%)(median, range)** | 76 (42 – 106) | - |

**Supplementary Figure 1.** Plasma concentration-time curves of imatinib and imatinib-d8 following oral (p.o.) administration of 400 mg imatinib dose at t=0h and intravenous (i.v.) administration of a 100 µg imatinib-d8 microdose at t=2.5h in patients (1-6, **A-F**) displaying steady state imatinib plasma pharmacokinetics.
